# A Hidden Guardian: The Stability and Spectrum of Antibody-Dependent Cell-Mediated Cytotoxicity in COVID-19 Response in Chinese Adults

**DOI:** 10.3390/vaccines13030262

**Published:** 2025-02-28

**Authors:** Jinge Cao, Mengze Gan, Zhihao Zhang, Xiaosong Lin, Qi Ouyang, Hui Fu, Xinyue Xu, Zhen Wang, Xinlian Li, Yaxin Wang, Hao Cai, Qing Lei, Li Liu, Hao Wang, Xionglin Fan

**Affiliations:** 1 Department of Pathogen Biology, School of Basic Medicine, Tongji Medical College and State Key Laboratory for Diagnosis and Treatment of Severe Zoonotic Infectious Diseases, Hubei Key Laboratory of Drug Target Research and Pharmacodynamic Evaluation, Huazhong University of Science and Technology, Wuhan 430030, China; jingecao@126.com (J.C.); ganmengze@126.com (M.G.); d202081480@hust.edu.cn (X.L.); ouyangqi96@163.com (Q.O.); husterfuhui@163.com (H.F.); xxy718221908@163.com (X.X.); 2 Ministry of Education and State Key Laboratory of Environmental Health (Incubating), School of Public Health, Huazhong University of Science and Technology, Wuhan 430030, China; m202275468@hust.edu.cn (Z.Z.); d202181621@hust.edu.cn (Z.W.); lixinlian198754@163.com (X.L.); wangyaxin9987@163.com (Y.W.); caihao9797@163.com (H.C.); liul2012@hust.edu.cn (L.L.); 3 Division of Nephrology, Tongji Hospital, Tongji Medical College, Huazhong University of Science and Technology, Wuhan 430030, China; leiqinghust@163.com

**Keywords:** SARS-CoV-2, neutralizing antibodies, ADCC

## Abstract

Objectives: Identifying immune-protective biomarkers is crucial for the effective management and mitigation of current and future COVID-19 outbreaks, particularly in preventing or counteracting the immune evasion exhibited by the Omicron variants. The emergence of SARS-CoV-2 variants, especially those within the Omicron lineage, has highlighted their capacity to evade neutralizing antibodies, emphasizing the need to understand the role of antibody-dependent cell-mediated cytotoxicity (ADCC) in combating these infections. Methods: This study, conducted in Qichun City, Hubei province, from December 2021 to March 2023, involved 50 healthy Chinese adults who had received two doses of inactivated vaccines and had subsequently experienced mild infections with the Omicron BA.5 variant. Blood samples from these 50 healthy Chinese adults were collected at six distinct time points: at baseline and at the 1st, 3rd, 6th, and 9th months following the third dose of the inactivated vaccine, as well as 3 months post-breakthrough infection. Their sera were analyzed to assess ADCC and neutralization effects. Results: The results indicated that the antibodies elicited by the inactivated SARS-CoV-2 vaccine targeted the spike protein, exhibiting both pre-existing neutralizing and ADCC activities against Omicron variants BA.5 and XBB.1.5. Notably, the ADCC activity demonstrated greater stability compared to that of the neutralizing effects, persisting for at least 15 months post-vaccination, and could be augmented by additional vaccine doses and breakthrough infections. The ADCC effect associated with hybrid immunity effectively targets a spectrum of prospective Omicron variants, including BA.2.86, CH.1.1, EG.5.1, and JN.1. Conclusions: In light of its stability and broad-spectrum efficacy, we recommend the use of the ADCC effect as a biomarker for assessing protective immunity and guiding the development of vaccines and monoclonal antibodies.

## 1. Introduction

The persistent spread of SARS-CoV-2 Omicron variants continues to contribute to significant weekly mortality rates [1]. These variants have progressively accumulated mutations in the spike protein, enhancing their resistance to the neutralizing antibodies (nAbs) derived from vaccinations [2,3,4], breakthrough infections [5,6,7,8], and therapeutic monoclonal antibodies [3,9,10]. Therefore, it is imperative to devise more effective strategies to prevent or counteract the immune evasion demonstrated by Omicron variants in order to effectively manage and mitigate both current and future COVID-19 outbreaks.

The host’s adaptive immune response to viral infections encompasses both humoral and cellular components. Within the humoral response, antibodies are categorized as either neutralizing or non-neutralizing. nAbs that target the spike protein of SARS-CoV-2 can impede viral entry into host cells and neutralize free viruses in vivo [11]. Numerous studies have demonstrated that emerging SARS-CoV-2 Omicron variants have the capacity to evade nAbs [3,4,12,13,14] and the cellular immune responses [15,16] induced by vaccination. Nevertheless, vaccinated individuals experience lower rates of severe illness, hospitalization, and mortality upon infection with the earlier Omicron variants [17,18], indicating the presence of other immunological protective mechanisms that are not yet fully understood. Traditionally, non-neutralizing functions, such as antibody-dependent cell-mediated cytotoxicity (ADCC), have been considered crucial for the control and clearance of viral infections, including those caused by influenza [19,20,21,22], Ebola [23,24,25], and HIV [26,27,28,29,30]. Recent research has indicated that anti-spike antibodies in individuals recovering from an infection with a SARS-CoV-2 prototype strain (PS) or those vaccinated with an inactivated vaccine exhibit ADCC activity [31,32,33,34,35]. Furthermore, in individuals who have been vaccinated and subsequently infected with the Omicron BA.1 variant, their ADCC activity may exhibit greater longevity compared to that of the neutralizing effect [36]. It is urgent to comprehend the role of ADCC in preventing and mitigating the severity of the infections caused by emerging SARS-CoV-2 Omicron variants.

The diverse origins of the anti-spike antibodies in individuals infected during the COVID-19 pandemic, alongside breakthrough infections from various viral variants post-vaccination, present challenges in investigating the persistence and comprehensive effects of ADCC. Until 7 December 2022, China maintained a zero-COVID-19 policy, which embraced mass testing, social distancing, mask mandates, and vaccinations [37,38,39]. Mass vaccination efforts commenced in March 2021, and by January 2023, over 90% of the population had received the basic protocol with two vaccine doses, with more than 50% having received a third vaccine dose [40]. China’s pandemic prevention policy has facilitated the establishment of a consistent and well-defined immunological landscape against COVID-19 within its population. In a study conducted from December 2021 to March 2023, we established a cohort of healthy Chinese adults who had a clear history of vaccination and subsequently exposure to the Omicron BA.5 variant with mild illness. We analyzed their serum samples to explore the dynamics of the ADCC effect and its cross-reactivity with the prospective Omicron variants. Our findings suggest that the ADCC effect mediated by anti-spike antibodies demonstrates greater stability and a broader range of activity than those of the neutralizing effect, indicating its potential as an alternative biomarker for protective immunity.

## 2. Methods

### 2.1. The Study Cohort

This study involved 400 healthy adults from Qichun City, Hubei province, who had received two doses of the inactivated vaccine six months prior and were initially uninfected with SARS-CoV-2. They then received a third vaccine dose and had later been infected with the Omicron BA.5 variant. Their blood samples were collected at the baseline; 1, 3, 6, and 9 months post their third vaccine dose; and 3 months post-breakthrough infection. This study established comprehensive exclusion parameters, including the following: immunocompromised status (self-reported), previous SARS-CoV-2 exposure, undocumented vaccination status, hypersensitivity to vaccine components, recent febrile illness (≤14 days), blood product administration within the past 120 days, and protocol non-compliance. The participant retention decreased progressively during the study period, culminating in a final cohort of 50 subjects by the sixth study visit. To ensure the availability of complete longitudinal data, these 50 participants with complete follow-up data were chosen for analysis. The cohort study was established between December 2021 and March 2023.

### 2.2. The Cell Lines

HEK 293T cells (ATCC CRL-3216, Manassas, VA, USA) and Vero E6 cells (ATCC CRL-1587) were grown in DMEM (Gibco, Thermo Fisher Scientific, Waltham, MA, USA) with 10% FBS (Biological Industries, Kibbutz Beit Haemek, Israel) and 100 U/mL penicillin–streptomycin (Biosharp, Labgic Technology, Beijing, China). The Jurkat-CD16-NFAT-Luc cells (InvivoGen, San Diego, CA, USA) for the ADCC bioassay were cultured in RPMI 1640 (Gibco) with 20% FBS, 100 U/mL penicillin–streptomycin, 200 μg/mL hygromycin (Solarbio Science & Technology, Beijing, China), and 2 μg/mL puromycin (Yeasen Biotechnology, Shanghai, China). All of these cells were cultured at 37 °C with 5% CO_2_.

### 2.3. The ADCC Assay

The full-length genes encoding the spike proteins of the SARS-CoV-2 PS and various Omicron variants were commercially synthesized using eukaryotic codon optimization and subsequently cloned into the eukaryotic expression vector pVAX-1. HEK-293T cells overexpressing the SARS-CoV-2 spike protein were generated as the target cells following transfection with the resulting recombinant plasmids and were verified in accordance with the methodology outlined in our previous study [41]. After 24 h of culture at 37 °C with 5% CO_2_, the cells were harvested and subjected to immunostaining using a spike-specific mAb (Sino Biological, Beijing, China). Detection was performed using a FITC-conjugated Goat Anti-Rabbit IgG secondary antibody (BD Biosciences, Franklin Lakes, NJ, USA). The fluorescence signals were analyzed using a flow cytometer (CytoFLEX, Beckman Coulter, Brea, CA, USA).

The Jurkat-CD16-NFAT-Luc cells were used as the effector cells to establish the ADCC detection platform. Before the ADCC assays, the serum dilution and the effector–target ratio were optimized. Twelve hours prior to the assay, 10^4^ target cells were placed per well. On the day of the assay, 100 μL of serum with 2-fold dilutions starting from 1:10 and ranging to 1:640 was added to each well and incubated with the target cells at 37 °C for 2 h. After removing the supernatant, the cells were washed with PBS to remove unbound antibodies. Then, 100 μL of the ADCC effector cells was added at varying ratios (1:1 to 8:1). The negative controls had no serum, and RPMI 1640 medium served as the background. After 12 h of incubation at 37 °C, the relative light units (RLU) were measured using the Bright-Glo™ Luciferase Assay System (Promega Corporation, Madison, WI, USA). The ADCC fold change was calculated using the formula (RLU of the experimental well—RLU of the background well)/(RLU of the control well with no serum—RLU of the background well).

### 2.4. Pseudovirus Neutralization Assays

Pseudovirus neutralization assays were conducted to measure the serum nAb titers against the spike proteins of the PS and Omicron variants BA.5, XBB.1.5, and JN.1 with an 18-amino-acid C-terminal truncation, as previously described [42]. The VSV-based pseudoviruses were produced using pCAGGS-SARS-CoV-2-spike Δ18 plasmids in the HEK 293T cells. The serum samples were diluted, mixed with the pseudovirus, and incubated with the Vero E6 cells. The luminescence was measured, and the results were expressed as NT50.

### 2.5. Statistical Analysis

Statistical analysis and visualization were performed using GraphPad Prism 9 and R version 4.3.3 software. Normality and lognormality tests were used to assess the normality of the distribution. When required, the Wilcoxon matched-pairs signed rank test and Spearman’s correlation were used, with *p* < 0.05 considered significant.

## 3. Results

### 3.1. The Dynamics of the nAb Responses to the SARS-CoV-2 PS and Omicron BA.5

**Figure 1:**
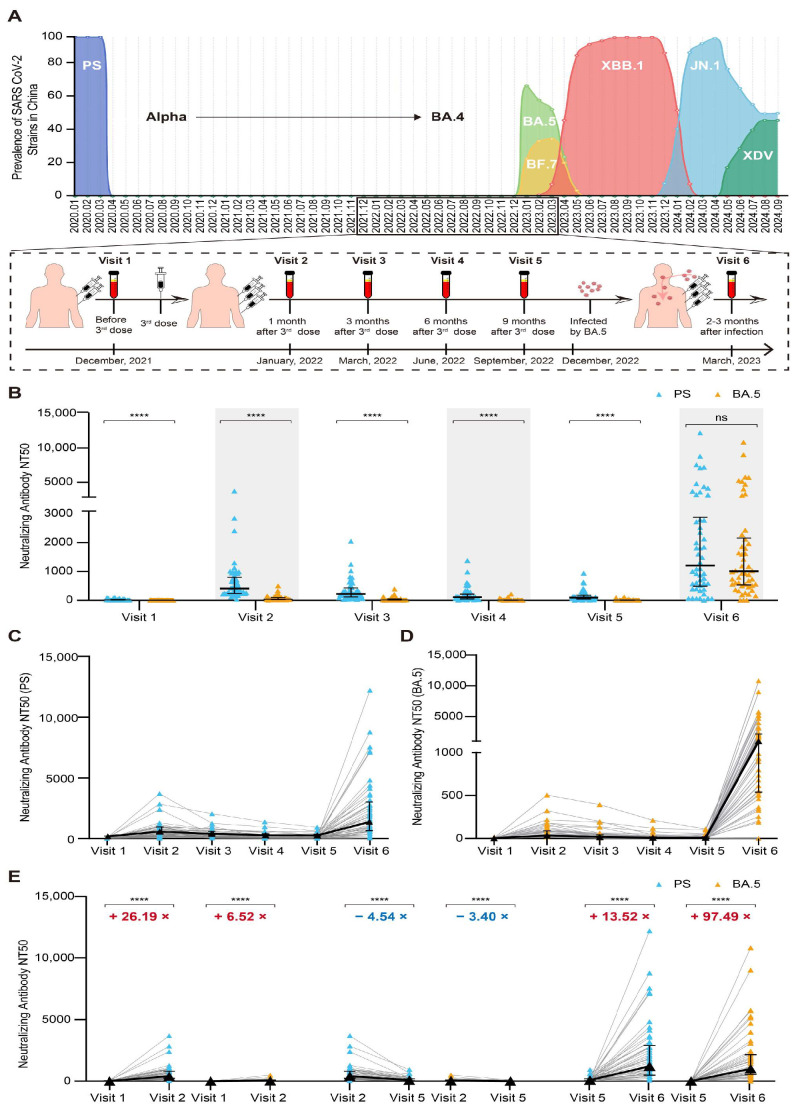
Temporal dynamics of nAb responses against the SARS-CoV-2 PS and Omicron BA.5. (**A**) The prevalence of SARS-CoV-2 strains in China during the pandemic, alongside the study cohort’s nAb responses, was evaluated at six distinct time points: at baseline (visit 1, 6 months following the administration of two doses of the inactivated vaccine) and at 1 month (visit 2), 3 months (visit 3), 6 months (visit 4), and 9 months (visit 5) after the administration of a third dose of the inactivated vaccine, as well as 3 months subsequent to a breakthrough infection (visit 6). (**B**) The levels of nAbs against the PS and the BA.5 variant were compared at different visits (N = 50). (**C**) Dynamic changes in nAbs against the PS (N = 50). (**D**) Dynamic changes in nAbs against the BA.5 variant (N = 50). (**E**) Comparative analysis of the nAbs before and after the third dose of the inactivated vaccine, at 1 month and 9 months post the third dose, and pre- and post-breakthrough infection (N = 50). The numbers highlighted in red denote significant increases in the fold changes, while those in blue indicate significant decreases in the fold changes. **** *p* < 0.0001.

Since the onset of the SARS-CoV-2 pandemic in January 2020, China’s dynamic zero-COVID-19 policy effectively maintained low infection rates for the SARS-CoV-2 PS and variants such as α, β, γ, δ, and Omicron subvariants BA.1, BA.2, BA.3, and BA.4. However, the termination of this policy in late 2022 resulted in an increase in infections of Omicron BA.5/BF.7, subsequently followed by the emergence of the Omicron variants XBB.1.5 and JN.1. A cohort study conducted from December 2021 to March 2023 selected 50 individuals who had received inactivated vaccines and had been infected with the Omicron BA.5 variant; all of the participants exhibited mild illness (Figure 1A). The median age of the participants was 50 years, with a gender distribution of 42% male and 58% female.

The serum nAb responses to the SARS-CoV-2 PS and the Omicron BA.5 variant were compared at all study visits (Figure 1B). Across all visits, with the exception of the final visit involving breakthrough infections, the levels of nAbs against the PS were significantly higher than those against the Omicron BA.5 variant. Furthermore, the temporal dynamics of the nAb responses to both the PS (Figure 1C) and the Omicron BA.5 variant (Figure 1D) demonstrated similar patterns throughout the experimental period. One month following the administration of the third vaccine dose, the levels of nAbs against the PS exhibited a substantial 26.19-fold increase (Figure 1E) compared to the pre-vaccination levels, while a slight increase in the titers of the nAbs against the Omicron BA.5 variant was also observed; however, 9 months after the third vaccine dose, these levels declined significantly 3.4 to 4.54-fold over time (PS: 407.055 vs. 89.625; Omicron BA.5: 35.115 vs. 10.34). Notably, breakthrough infections strongly raised the levels of the nAbs (PS: 89.625 vs. 1211.77; Omicron BA.5: 10.34 vs. 1008.02; Figure 1E). Consequently, prior to the Omicron BA.5 pandemic, nAb responses to this variant had been established in their serum through vaccination. Both the administration of the third vaccine dose and the occurrence of breakthrough infections resulted in a substantial increase in their levels of nAbs against both the PS and the BA.5 variant. Extending the post-vaccination period to nine months was associated with a significant reduction in nAb levels, which resulted in breakthrough infections.

### 3.2. The Establishment of the ADCC Detection Technology Platform

**Figure 2:**
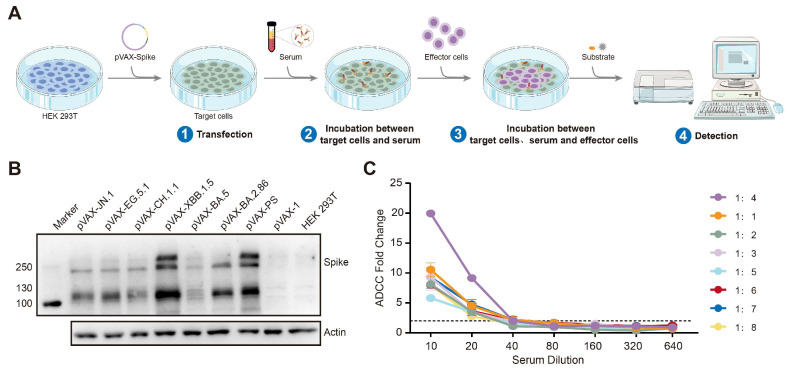
Establishment of the ADCC detection technology platform. (**A**) The processes involved in the ADCC assay. (**B**) The expression of the spike protein in the cell lysates transfected with various recombinant eukaryotic expressing plasmids, including pVAX-PS, pVAX-BA.2.86, pVAX-BA.5, pVAX-XBB.1.5, pVAX-CH.1.1, pVAX-EG.5.1, and pVAX-JN.1. Cells transfected with the pVAX-1 plasmid and HEK 293T cells served as the controls. (**C**) The optimal serum dilution and effector-to-target ratio for the ADCC assays. Cells transfected with pVAX-PS as the target cells and serum samples from ten individuals were pooled for testing purposes.

In this study, an advanced platform for the detection of ADCC was successfully developed, with the assay procedure detailed in Figure 2A. This platform incorporates recombinant eukaryotic expression plasmids encoding the spike proteins of the SARS-CoV-2 PS and Omicron variants, including BA.2.86, BA.5, XBB.1.5, CH.1.1, EG.5.1, and JN.1. These plasmids are transfected into HEK 293T cells, which serve as the target cells, while the Jurkat-Lucia-NFAT-CD16 cell line is employed as the effector cells. To verify the expression of the spike proteins in the HEK 293T cells, cell lysates were subjected to a Western blot analysis, revealing the prominent expression of the full-length spike protein at approximately 180 kDa, as illustrated in Figure 2B. The cell surface expression levels of the spike proteins were evaluated using flow cytometry (Figure S1). A serum dilution of 1:20 was required to achieve an ADCC fold change of 2, with the optimal effector-to-target cell ratio determined to be 4:1, maximizing the ADCC fold change with 10^4^ target cells and 4 × 10^4^ effector cells per well in 96-well plates (Figure 2C). This platform was subsequently utilized to assess the ADCC response against the spike proteins of the PS and Omicron BA.5, XBB.1.5, and other variants in the cohort sera.

### 3.3. Dynamics of the ADCC Responses to the SARS-CoV-2 PS and Omicron BA.5

**Figure 3:**
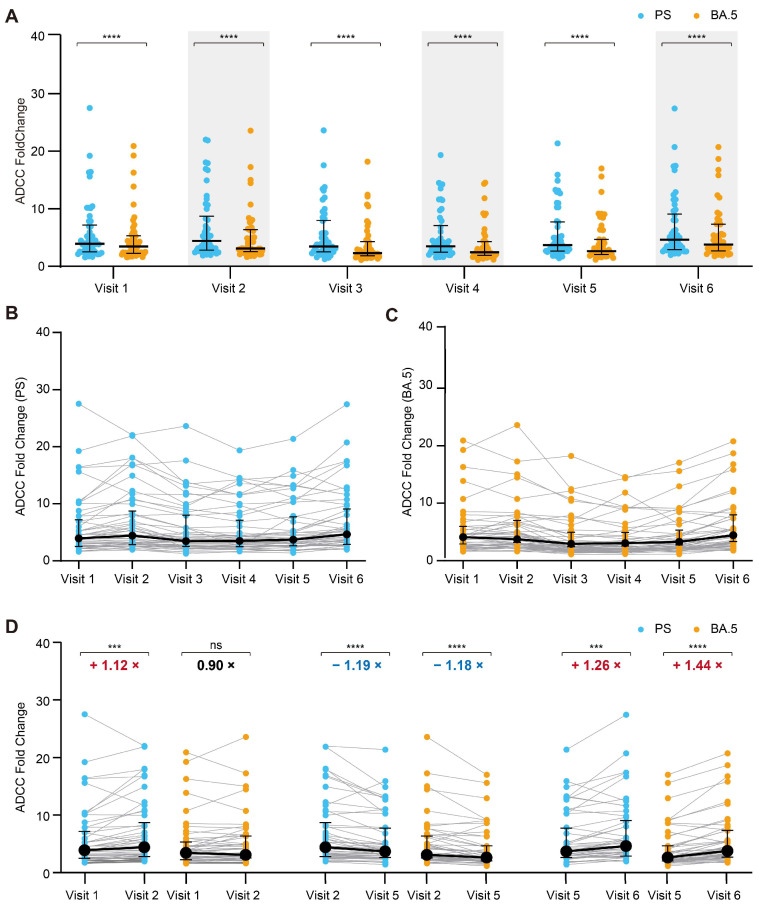
Temporal dynamics of the ADCC responses against the SARS-CoV-2 PS and Omicron BA.5. (**A**) ADCC levels against the PS and BA.5 variants were compared at different visits (N = 50). (**B**) Dynamic changes in ADCC against the PS (N = 50). (**C**) Dynamic changes in ADCC against the BA.5 variant (N = 50). (**D**) Comparative analysis of ADCC before and after the third dose of the inactivated vaccine at 1 month and 9 months post the third dose, and pre- and post-breakthrough infection (N = 50). The numbers highlighted in red denote significant increases in the fold changes, while those in blue indicate significant decreases in the fold changes. *** *p* < 0.001; **** *p* < 0.0001.

Our study demonstrated that the ADCC effect against the spike protein of the PS was consistently stronger than that against the BA.5 variant at all of the time points assessed (Figure 3A). Furthermore, the temporal patterns in the ADCC effect against both the PS (Figure 3B) and the BA.5 variant (Figure 3C) exhibited similar trends. An in-depth analysis of the impact of the third vaccine dose, the duration following vaccination, and breakthrough infection on the ADCC responses was performed. One month subsequent to the administration of the third vaccine dose, there was a modest increase in the ADCC activity against the spike protein of the PS (Figure 3D), whereas the third vaccine dose did not enhance the ADCC activity against the Omicron BA.5 variant. At the ninth month following the third dose, there was a slight reduction in the ADCC activity of the antibodies against the spike proteins of both the PS and the BA.5 variant (PS: 4.408 vs. 3.703; Omicron BA.5: 3.099 vs. 2.635; Figure 3D). Three months following a breakthrough infection, a slight increase in the ADCC activity against both spike proteins was observed (PS: 3.703 vs. 4.650; Omicron BA.5: 2.635 vs. 3.805; Figure 3D). Therefore, these pre-existing antibodies present in the serum of individuals immunized with vaccines exhibited potential ADCC effects against the prospective Omicron variants, and spike-protein-specific antibodies with ADCC activity against the Omicron variant were sustained in the cohort’s sera for a minimum of 15 months following the administration of the second vaccine dose.

### 3.4. The ADCC Effect Against the Prospective Omicron Variant XBB.1.5

**Figure 4:**
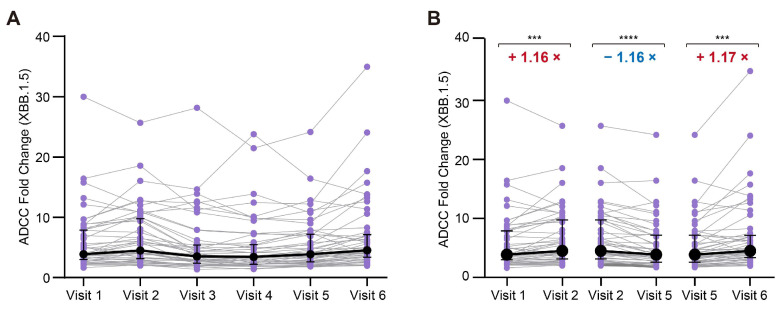
Temporal dynamics of the ADCC responses to Omicron XBB.1.5. (**A**) Dynamic changes in ADCC against the XBB.1.5 variant (N = 50). (**B**) Comparative analysis of ADCC against the XBB.1.5 variant before and after a third dose of the inactivated vaccine at 1 month and 9 months post the third dose and pre- and post-breakthrough infection (N = 50). The numbers highlighted in red denote significant increases in the fold changes, while those in blue indicate significant decreases in the fold changes. *** *p* < 0.001; **** *p* < 0.0001.

Subsequent to the cohort coverage period, we conducted an additional assessment of the ADCC effect of serum antibodies targeting the other prospective Omicron variant XBB.1.5 to confirm our findings (Figure 4). Interestingly, the ADCC dynamics against the variant XBB.1.5 were similar to those observed for the PS (Figure 4). One month after the third vaccine dose, there was a significant enhancement in the ADCC effect against XBB.1.5 when compared to that pre-administration. At the nine month following the third dose, there was a slight reduction in the ADCC activity of the antibodies against the spike protein of XBB.1.5 (3.099 vs. 2.635; Figure 4B). Notably, a slight increase in the ADCC activity was observed 3 months following a breakthrough infection (2.635 vs. 3.805; Figure 4B). Therefore, the ADCC effect against the prospective variant XBB.1.5 had also been established, persisted for up to 15 months in the serum samples after the second vaccine dose, and extended to at least 18 months after a breakthrough infection with BA.5.

### 3.5. The Broad-Spectrum Activity of ADCC Against the Prospective Circulating Omicron Variants

**Figure 5:**
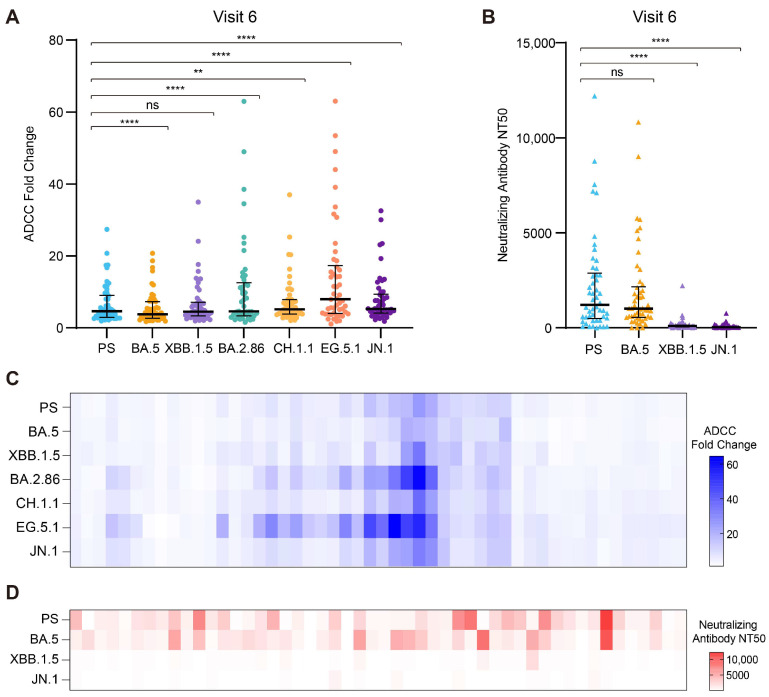
Analysis of the ADCC effect and the neutralizing activity of serum samples collected at visit 6 for a range of prospective Omicron variants. (**A**) Comparison of the ADCC effect of serum samples from visit 6 against the PS and Omicron variants BA.5, XBB.1.5, BA.2.86, CH.1.1, EG.5.1, and JN.1 (N = 50). (**B**) Comparison of nAb responses of serum samples from visit 6 to the PS and Omicron variants BA.5, XBB.1.5, and JN.1 (N = 50). (**C**) The ADCC effect of individual serum samples from visit 6 against the same set of Omicron variants (N = 50). (**D**) Neutralizing activity of individual serum samples from visit 6 against the same set of Omicron variants (N = 50). ** *p* < 0.01; **** *p* < 0.0001.

To investigate the impact of breakthrough infections with the BA.5 variant, we conducted a comprehensive assessment of the ADCC effect and the neutralizing activity of the serum samples with hybrid immunity collected at visit 6. These samples were tested against a range of prospective Omicron variants, extending beyond the cohort’s initial coverage period. Interestingly, the antibodies present in the visit 6 sera demonstrated an ADCC effect against the spike proteins of these Omicron variants (Figure 5A). Notably, the ADCC effect was particularly pronounced against the spike proteins of the BA.2.86, CH.1.1, EG.5.1, and JN.1 variants when compared to that for the PS. Although the nAb responses in the serum samples collected at visit 6 to the spike protein of the PS and the BA.5 variant were comparable, the cohort demonstrated reduced nAb levels in response to the XBB.1.5 and JN.1 variants. The ADCC effect (Figure 5C) and nAb response (Figure 5D) of the individual serum samples for various Omicron variants are depicted in the heatmap, which supports these observations. Consequently, breakthrough infections with a specific variant primarily induce high levels of nAbs against the infecting variant and previously circulating strains while demonstrating limited cross-neutralization activity against emerging Omicron variants. In contrast, the ADCC effect in the context of hybrid immunity exhibits broad-spectrum efficacy against a wide range of prospective Omicron variants.

### 3.6. The Correlation Between the ADCC Effect and Neutralizing Activity

**Figure 6:**
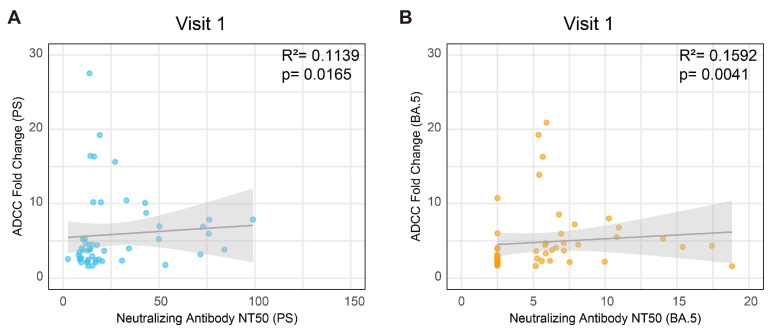
Correlation between ADCC and the neutralizing effect against the PS and the Omicron BA.5 variant at visit 1. Correlation between ADCC and the neutralizing effect against the PS (**A**) and the Omicron BA.5 variant (**B**) at visit 1. R represents Spearman’s correlation coefficient (N = 50).

Given that the ADCC effect and the neutralizing activity are mediated by the same anti-spike antibody, their relationship was systematically evaluated across all visits. During the initial visit, conducted six months post-administration of the second dose of the inactivated vaccine, a weak correlation was observed between the ADCC effect and the nAb titer against both the PS (Figure 6A) and the BA.5 variant (Figure 6B). However, in subsequent visits or against different variants, no significant correlation was identified (Supplementary Figure S2A–L). These findings suggest distinct mechanistic functions between the ADCC effect and the neutralizing activity.

## 4. Discussion

This study investigated a cohort of Chinese adults with a documented history of vaccination and exposure to SARS-CoV-2 who experienced mild infections to elucidate the dynamics and cross-reactive activity of the ADCC effect of anti-spike antibodies in the serum both prior to and following the administration of a third vaccine dose, as well as after a breakthrough infection. Our research demonstrated that the pre-existing antibodies elicited in the serum of the Chinese individuals vaccinated with an inactivated SARS-CoV-2 PS vaccine were capable of targeting the spike protein, exhibiting both neutralizing effects and ADCC activities against the prospective circulating Omicron variants. Notably, this study reveals that the broad-spectrum ADCC activity against the prospective Omicron variants was more stable than the neutralizing effect and persisted in the cohort’s serum for at least 15 months following basic vaccination protocols. This ADCC activity could be enhanced through both booster inoculations and breakthrough infections, thus further extending its duration.

Our study demonstrated that the BA.5 variant exhibited a significant immune evasion capability against the nAbs elicited by the administration of a third vaccine dose, aligning with the findings of previous research [5,43,44]. Concurrently, multiple studies have corroborated that various Omicron subvariants, including XBB.1 [4,45,46,47], BQ.1 [4,45,46,47], EG.5.1 [48,49,50], BA.2.86 [49,50,51,52], and JN.1 [49,50,52,53], similarly possess a robust capacity to evade the nAbs induced by either vaccination or natural infection. This immune escape is attributed to the high variability observed in the spike proteins of these variants, which compromises the protective efficacy of pre-existing nAbs in the serum [5,54]. Nevertheless, all of the participants in this study had only experienced mild infections after breakthrough infections with the BA.5 variant. This suggests the protective role of non-neutralizing antibody effects in mitigating severe COVID-19 outcomes. In the context of HIV-1, individuals with high antibody titers exhibiting ADCC effects are less likely to progress to acquired immune deficiency syndrome compared to those with lower antibody titers [55]. The HIV-1 vaccine RV144 confers protection by inducing non-neutralizing antibodies that exhibit ADCC effects [56,57,58]. Similarly, monoclonal antibodies targeting the influenza virus, such as 651 [59], 38C2 [60], and cHA [61], primarily mediate protection through the ADCC mechanism. Collectively, these studies underscore the pivotal role of ADCC in the prevention and management of these viral infections. Previous research has demonstrated that various vaccines and infections can induce ADCC activity against the spike protein of SARS-CoV-2 and its variants, including Omicron variants BA.1, BA.2, BA.5, and XBB.1.5, which exhibit only a slight evasion of this effect compared to the neutralization activity [34,36,62,63,64,65]. The non-neutralizing monoclonal antibody C10 has been shown to mediate ADCC, effectively reducing viral titers by targeting infected cells in the lungs [66]. Similarly, the CV804 monoclonal antibody, despite lacking neutralizing activity, exhibits a strong ADCC effect that can inhibit the disease progression in animal models of SARS-CoV-2 infection [67]. Furthermore, the ADCC response has been observed to persist for at least 26 weeks following the administration of a third dose of an inactivated vaccine [34]. However, these studies have not fully elucidated the protective role of the ADCC effect or its broad-spectrum activity. In this study, we established a cohort of Chinese adults and developed a platform to assess ADCC effects, aiming to investigate the dynamic changes in the ADCC activity against the spike proteins of various Omicron variants during vaccination and infection. We selected HEK 293T cells transfected with recombinant eukaryotic expression plasmids as the target cells and employed the Jurkat-NFAT-Luc cell line as the effector cells to establish an ADCC effect assay platform. Compared to alternative methodologies, this platform is widely utilized and offers advantages such as enhanced experimental repeatability and reduced variability [68,69]. The resulting data facilitate inter-laboratory comparisons, as well as comparisons across different experimental batches and samples from diverse sources, thereby supporting the integration of ADCC effects into the index system for evaluating the efficacy of vaccines and monoclonal antibodies. Our study further elucidates that the pre-existing ADCC effect can persist for up to at least 15 months, exhibiting its broad-spectrum activity against multiple prospective Omicron variants, including BA.2.86, CH.1.1, EG.5.1, and JN.1. Additionally, we identified a weak correlation between the ADCC effects and the neutralizing antibody levels six months following the second vaccine dose, consistent with previous research [70,71]. However, this correlation diminished following the administration of a third vaccine dose and subsequent breakthrough infections, possibly due to the incomplete overlapping epitopes [72].

The present study is constrained by several limitations that warrant acknowledgment. In order to compare the effects between the neutralizing and ADCC effects, our ADCC measurements were concentrated on the spike protein, which may have led to oversight of the immunological significance of other viral components. Furthermore, the restriction of our study cohort to individuals with mild symptoms limited the ability to conduct a meaningful analysis of potential associations between the clinical disease severity and ADCC responses, which remain to be investigated.

In short, given the stability, persistence, broad-spectrum activity, and significant role of the ADCC effect in protective immunity against infections, we propose that the ADCC effect assay should be utilized as an alternative biomarker for assessing protective immunity within the defined population and informing the development of effective vaccines and monoclonal antibodies for combating future Omicron variant outbreaks.

## 5. Conclusions

The ADCC effect targeting the spike protein of SARS-CoV-2 is more stable than the neutralization effect and has broad-spectrum efficacy against multiple prospective Omicron variants. We recommend the use of the ADCC effect as a biomarker to assess protective immunity and guide vaccine and monoclonal antibody development.

## Data Availability

The data that support the findings of this study are available from the corresponding author upon reasonable request.

## Supplementary Materials

The following supporting information can be downloaded at: https://www.mdpi.com/article/10.3390/vaccines13030262/s1, Figure S1: Expression levels of spike proteins from different variants on the surface of transfected HEK 293T Cells; Figure S2: Correlation between ADCC and neutralizing effect against PS, Omicron BA.5 variant, Omicron XBB.1.5 variant and Omicron JN.1 variant.
