# Supplementary material for: A Hidden Guardian: The Stability and Spectrum of Antibody-Dependent Cell-Mediated Cytotoxicity in COVID-19 Response in Chinese Adults
Source: Vaccines (Basel). 2025 Feb 28;13(3):262. doi: 10.3390/vaccines13030262 (PMC11945335; doi:10.3390/vaccines13030262)

Supplement

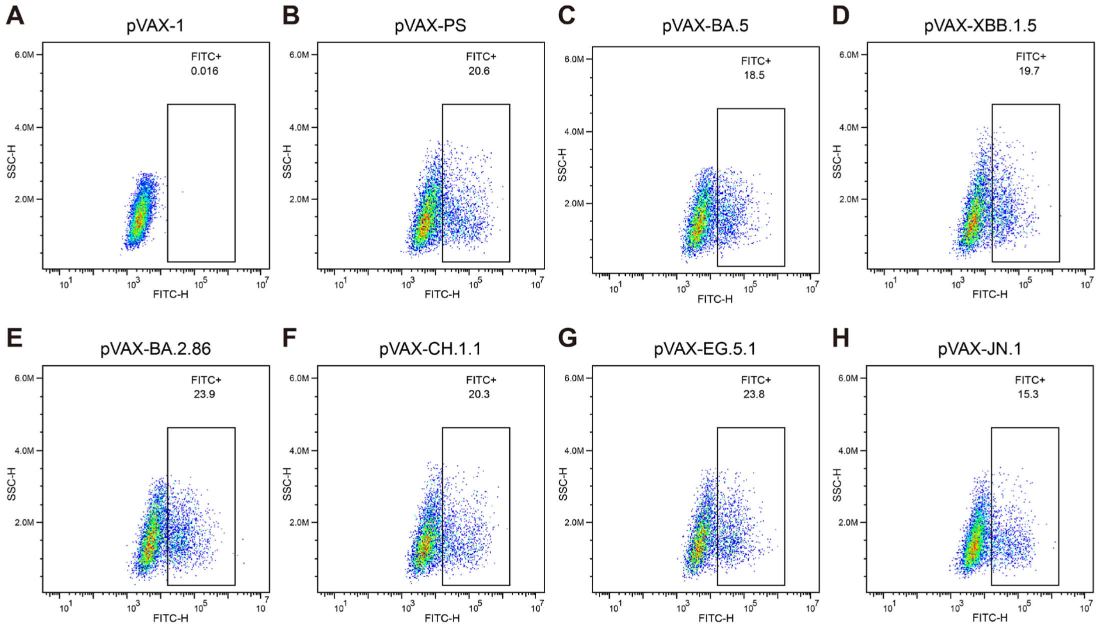

Figure S1. Expression levels of spike proteins from different variants on the surface of transfected HEK 293T Cells

The cell surface expression levels of spike proteins were evaluated using flow cytometry. The FITC+ value displayed in the upper right corner indicates the percentage of cells expressing the spike protein.

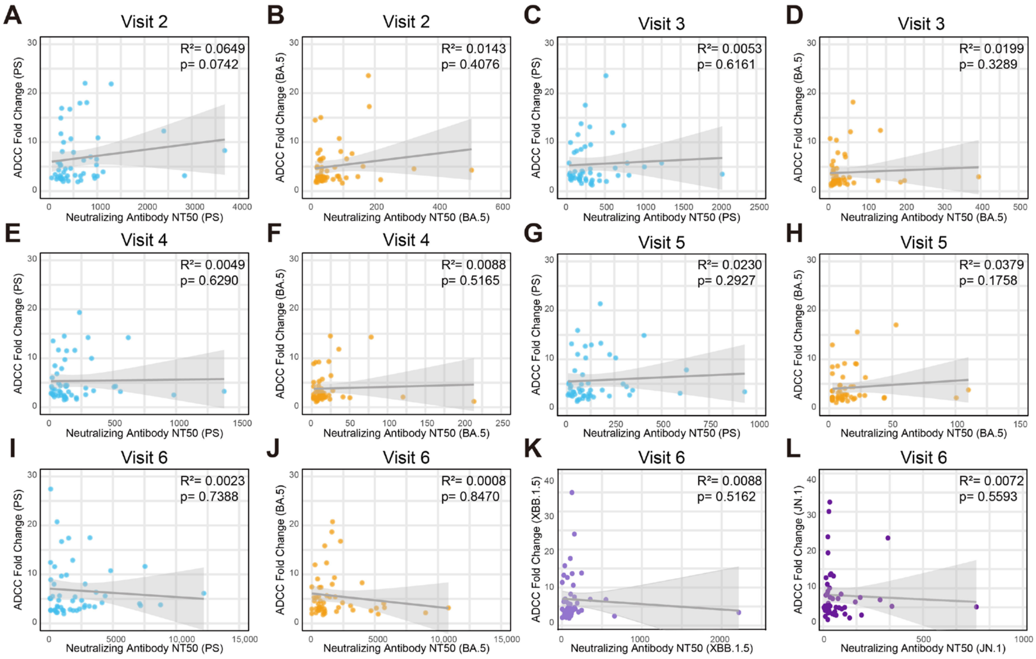

Figure S2. Correlation between ADCC and neutralizing effect against PS, Omicron BA.5 variant,

Omicron XBB.1.5 variant and Omicron JN.1 variant.

Correlation between ADCC and neutralizing effect against PS (A, C, E, G, I), Omicron BA.5 (B, D, F, H, J) variant, Omicron XBB.1.5 (K) variant and Omicron JN.1(L) variant. R represents the Spearman correlation coefficient (N=50).

Original images of western blots

Figure 2B: Spike

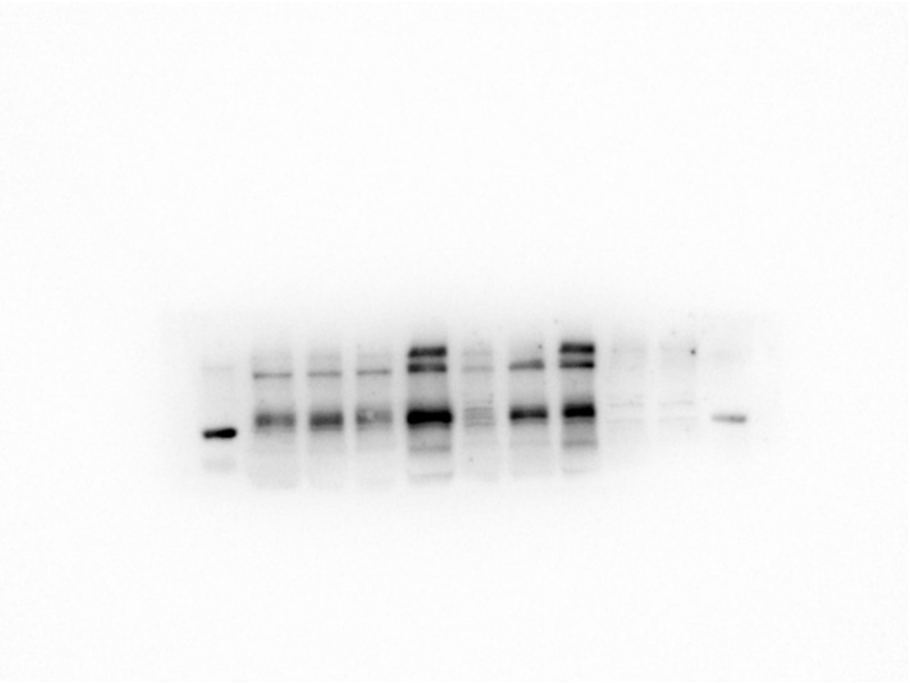

Figure 2B: Actin

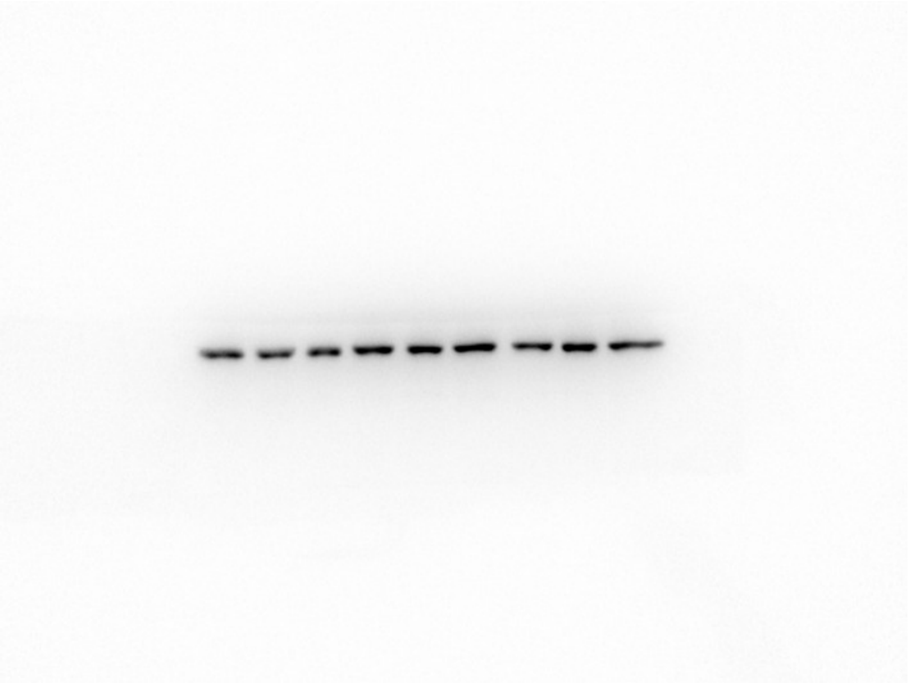

Supplement: Supplementary file 1 [file vaccines-13-00262-s001.zip › vaccines-3468370-supplementary.pdf]
